# Supplementary material for: SyNDI: synchronous network data integration framework
Source: BMC Bioinformatics. 2018 Nov 6;19:403. doi: 10.1186/s12859-018-2426-5 (PMC6219086; doi:10.1186/s12859-018-2426-5)
Supplement: Supplementary file 5 — Files of the example presented in Additional file 4. This additional file is a zip package that contains all files used in Additional file 4. Detailed descriptions of each of these files are presented in a README file included in this zip package. (GZ 30411 kb) [file 12859_2018_2426_MOESM5_ESM.gz › SyNDITools/Meme2Fimo/meme_4.9.0_4.tar.gz!/meme_4.9.0/doc/glam2mask_man.html]

glam2mask Manual


# glam2mask Manual

This program masks a glam2 motif out of sequences, so that weaker
motifs can be found. Masking replaces residues aligned to key positions
with the symbol 'x'. By alternately applying glam2 and glam2mask several
times, it is possible to find the strongest, second-strongest,
third-strongest, etc. motifs in a set of sequences.

- Mask the top motif in my\_motif.glam2 out of my\_seqs.fa, and
  store the result in mask1.fa:

  ```
  glam2mask -o mask1.fa my_motif.glam2 my_seqs.fa
  ```
- Mask the top motif in my\_motif.glam2 out of my\_seqs.fa, using
  'n' as the mask symbol:

  ```
  glam2mask -x n my_motif.glam2 my_seqs.fa
  ```

If the sequences do not have unique names, glam2mask may mask the
wrong ones. It also assumes that the order of the sequences does not
change, so don't do that.
